# Deep learning-based 3D cerebrovascular segmentation workflow on bright and black blood sequences magnetic resonance angiography

**DOI:** 10.1186/s13244-024-01657-0

**Published:** 2024-03-22

**Authors:** Langtao Zhou, Huiting Wu, Guanghua Luo, Hong Zhou

**Affiliations:** 1 School of Cyberspace Security, Guangzhou University, Guangzhou, 510006 China; 2 Department of Radiology of the First Affiliated Hospital of the University of South China, Hengyang, 421001 China

**Keywords:** Angiography, Black blood imaging, Cerebrovascular segmentation, Deep learning, Magnetic resonance

## Abstract

**Background:**

Cerebrovascular diseases have emerged as significant threats to human life and health. Effectively segmenting brain blood vessels has become a crucial scientific challenge. We aimed to develop a fully automated deep learning workflow that achieves accurate 3D segmentation of cerebral blood vessels by incorporating classic convolutional neural networks (CNNs) and transformer models.

**Methods:**

We used a public cerebrovascular segmentation dataset (CSD) containing 45 volumes of 1.5 T time-of-flight magnetic resonance angiography images. We collected data from another private middle cerebral artery (MCA) with lenticulostriate artery (LSA) segmentation dataset (MLD), which encompassed 3.0 T three-dimensional T1-weighted sequences of volumetric isotropic turbo spin echo acquisition MRI images of 107 patients aged 62 ± 11 years (42 females). The workflow includes data analysis, preprocessing, augmentation, model training with validation, and postprocessing techniques. Brain vessels were segmented using the U-Net, V-Net, UNETR, and SwinUNETR models. The model performances were evaluated using the dice similarity coefficient (DSC), average surface distance (ASD), precision (PRE), sensitivity (SEN), and specificity (SPE).

**Results:**

During 4-fold cross-validation, SwinUNETR obtained the highest DSC in each fold. On the CSD test set, SwinUNETR achieved the best DSC (0.853), PRE (0.848), SEN (0.860), and SPE (0.9996), while V-Net achieved the best ASD (0.99). On the MLD test set, SwinUNETR demonstrated good MCA segmentation performance and had the best DSC, ASD, PRE, and SPE for segmenting the LSA.

**Conclusions:**

The workflow demonstrated excellent performance on different sequences of MRI images for vessels of varying sizes. This method allows doctors to visualize cerebrovascular structures.

**Critical relevance statement:**

A deep learning-based 3D cerebrovascular segmentation workflow is feasible and promising for visualizing cerebrovascular structures and monitoring cerebral small vessels, such as lenticulostriate arteries.

**Key points:**

• The proposed deep learning-based workflow performs well in cerebrovascular segmentation tasks.

• Among comparison models, SwinUNETR achieved the best DSC, ASD, PRE, and SPE values in lenticulostriate artery segmentation.

• The proposed workflow can be used for different MR sequences, such as bright and black blood imaging.

**Graphical Abstract:**

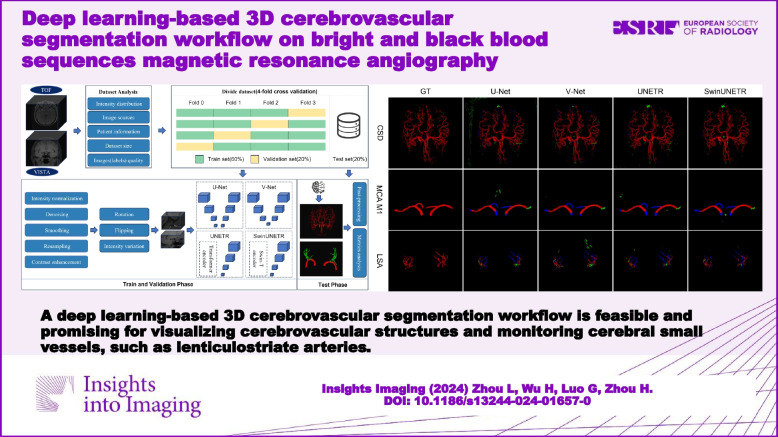

## Introduction

The incidence of cerebrovascular diseases, including cerebral venous thrombosis [1], has been increasing annually, surpassing incidence expectations. Several studies [2, 3] have indicated that risk factors such as hypertension and hyperglycemia are crucial factors in cerebrovascular diseases, including stroke. Due to lifestyle changes, patients are being diagnosed with these diseases at increasingly younger ages, underscoring the necessity of early screening and prevention for these conditions.

Magnetic resonance angiography (MRA) [4], a specific application of MRI, allows blood flow and both normal and diseased blood vessels to be visualized. MRA is notably noninvasive, making it a preferred early cerebrovascular disease screening method. Time-of-flight MRA (TOF-MRA) [5] enables contrast-media-free visualization of vascular morphology. Additionally, black blood imaging techniques [6], such as the three-dimensional T1-weighted sequence of volumetric isotropic turbo spin echo acquisition (3D T1 VISTA), exhibit high signal-to-noise ratios, high contrast, and anisotropic resolutions, enabling more precise imaging of small blood vessels, such as the lenticulostriate artery (LSA) [7].

Directly interpretating images still requires medical professionals to have high skill levels. A more modern approach involves segmenting and reconstructing the 3D structure of cerebral blood vessels. 3D reconstructions complement the interpretations of radiologists, and segmentation allows automated image analysis pipelines to be used. This enables doctors to directly observe lesions and vascular conditions and has numerous clinical applications such as identifying blood flow behavior, detecting tumors [8], aiding neurosurgical navigation [9], and designing and implementing cerebral vascular scaffolds [10]. Vascular diseases, particularly in the middle cerebral artery (MCA) and LSA, play a significant role in dementia, being major causes of Alzheimer’s disease [11]. These diseases can greatly contribute to cognitive decline and dementia, especially in patients older than 60 years. The MCA supplies blood to brain areas responsible for motor, sensory, and language functions, while the LSA supplies blood to the basal ganglia, which is essential for motor learning and cognitive functions [12]. Blockages in these arteries can lead to irreversible impairments and significantly affect quality of life. Therefore, precisely segmenting the MCA and LSA is key for effectively managing stroke and small vessel disease [13] and for improving patient neurological outcomes.

Over the past few decades, numerous experts have proposed classic methods for cerebrovascular segmentation, including thresholding-based [14], active contour model-based [15], and statistical model-based [16] segmentation methods. While these traditional techniques can yield good cerebrovascular segmentation results, they rely heavily on texture features in the image, sometimes even necessitating manual and delicate feature extraction. Moreover, with the increasing abundance of multimodal medical images, these traditional methods have become less competitive in image processing tasks.

Owing to the rapid development of deep learning technology, particularly in the computer vision field, various cerebrovascular segmentation methods based on deep learning have been proposed. Phellan et al. [17] pioneered deep convolutional neural networks for the cerebrovascular segmentation task. Shelhamer et al. [18] introduced a fully connected network that paved the way for end-to-end image segmentation. In the medical field, Ronneberger et al. proposed U-Net [19]. U-Net has since become a pivotal backbone network for end-to-end medical image segmentation tasks due to its symmetric lightweight encoder-decoder structure. Subsequently, several researchers [20, 21] have enhanced the U-Net’s design to achieve superior cerebrovascular segmentation. Moreover, Transformer [22], a deep learning model based on the self-attention mechanism, initially found extensive applications in natural language processing tasks such as machine translation and language modeling. Due to its powerful modeling capabilities, the transformer mechanism, including the Vision Transformer (ViT), has also achieved remarkable success in the computer vision field [23]. As a result, transformer-based cerebrovascular segmentation models [24, 25] have emerged. Although these models have been shown to be effective for TOF-MRA, they have never been validated on 3D T1 VISTA sequences. The methods have been focused on whole-brain vascular segmentation rather than smaller specific vascular segments, such as lenticulostriate arteries.

In this study, we aim to create a fully automated deep learning workflow that achieves accurate 3D segmentation of cerebral blood vessels. This is accomplished by combining classic CNN and transformer models and implementing standard data analysis, preprocessing, enhancement, modeling, and postprocessing techniques. Ultimately, we aim to adaptively reconstruct vascular structures in multisequence MR brain images.

## Materials and methods

The institutional review board approved this study (approval number 2021110623007), which was conducted according to the Helsinki Declaration. The researchers clearly informed the subjects about the experimental procedures. Their consent to voluntarily participate in the project was obtained, and signed informed consent forms were collected.

### Dataset

#### Cerebrovascular Segmentation Dataset (CSD)

Chen et al. [26] curated a cerebrovascular segmentation dataset, which is accessible at xzbai.buaa.edu.cn/datasets.html. The dataset comprises 45 volumes of TOF-MRA data obtained through 1.5 T GE MRI from the IXI dataset [27]. For accurate ground truth annotation, multiple radiologists, each with more than 3 years of clinical experience, meticulously labeled each volume with voxels. All the volumes were 1024 × 1024 × 92 in size, with a spatial resolution of 0.264 mm × 0.264 mm × 0.8 mm.

#### MCA M1 segment with LSA dataset (MLD)

We collected data from 107 patients, including outpatients and inpatients, at our hospital between 2014 and 2018. All 3D T1 VISTA images were 480 × 480 × 140 in size, with a spatial resolution of 0.4 mm × 0.4 mm × 0.4 mm. Voxel-level labeling of the image data was performed by a radiologist with more than three years of experience using ITK-SNAP. Additionally, the labeled MCA and LSA were thoroughly reviewed by another radiologist with more than ten years of experience. Physicians that processed the data were not aware of the clinical status of the patients to avoid bias.

Table 1 shows the details of the different datasets. N/A means not provided; note that 16 individuals did not provide sex information in the CSD.

**Table 1:** Details of different datasets. N/A means not provided; note that 16 individuals did not provide gender in the CSD

Information	CSD	MLD
Scanner	1.5 T GE	3.0 T Philips
Modality	TOF	3D T1 VISTA
TR/TE (ms)	26/4.2	400/19
Age (mean ± SD)	N/A	62 ± 11
Sex (F/M/NA)	20/9/16	42/65
Sum/Train/Val/Test	45/27/9/9	107/64/21/22
Resolution (mm^3^)	0.264 × 0.264 × 0.8	0.4 × 0.4 × 0.4
Volume size	1024 × 1024 × 92	480 × 480 × 140

*CSD* Cerebrovascular segmentation dataset, *MLD* Middle cerebral artery M1 segment with lenticulostriate artery segmentation dataset, *TOF* Time-of-flight, *3D T1 VISTA* Three-dimensional T1-weighted sequence of volumetric isotropic turbo spin echo acquisition

### Deep learning-based segmentation workflow

**Fig. 1:**
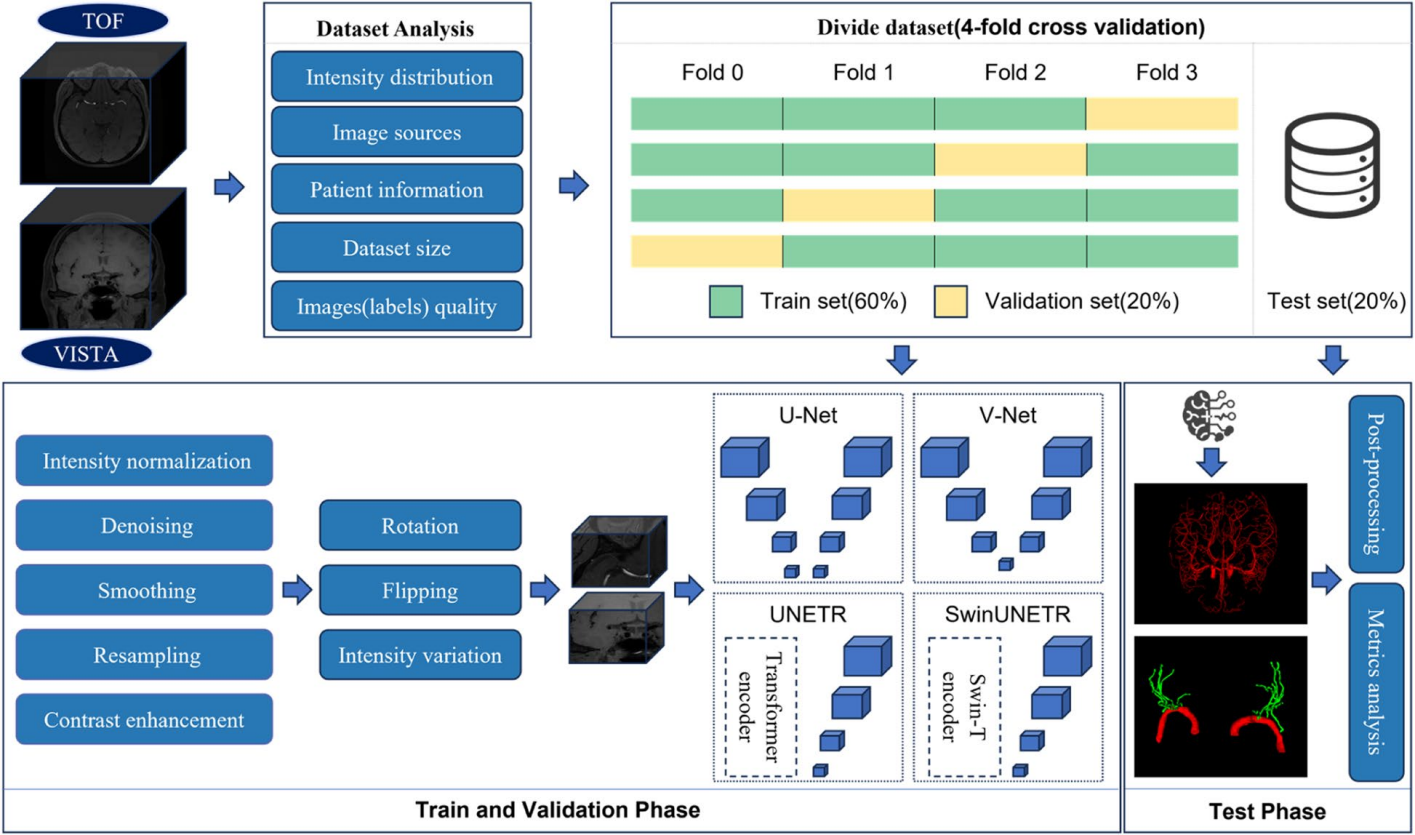
3D deep learning-based cerebrovascular segmentation workflow

The workflow encompasses five sequential steps: dataset analysis, data preprocessing, model training, model validation, and postprocessing with analysis. The overall flowchart is illustrated in Fig. 1.

### Dataset analysis

First, we thoroughly analyzed the dataset, focusing on assessing its overall size and the distributions of key variables. This analysis includes examining demographic distributions (such as age and sex), clinical characteristics (such as disease types and stages, scanning devices or protocols), and image feature diversity (such as size, shape, contrast, and intensity distribution). This analysis is conducted to gauge the dataset’s representativeness, detect biases or imbalances, and confirm the robustness and relevance of the findings to the target population or condition. We then evaluate factors such as image clarity, noise level, and the presence of artifacts. This involves assessing images against predefined criteria: for strong image clarity, images must exhibit sharp, well-defined edges and structures; for low noise levels, images must exhibit minimal random variations or “graininess”; finally, any distortions or anomalies that could interfere with accurate interpretation are defined as artifacts. Images failing to meet these standards, such as those with excessive noise or significant artifacts such as motion blur or ghosting, are excluded from the analysis to maintain the integrity and reliability of our dataset. Additionally, we verify the accuracy and consistency of the labels to ensure the cerebrovascular structures of interest are correctly labeled. Evaluating the dataset from these perspectives provides comprehensive insights into the characteristics, quality, and feasibility of the dataset. This process is designed to yield detailed statistical information about the dataset, guiding experimenters in critical aspects of cerebrovascular segmentation. Specifically, this information can be used to analyze the variabilities of thein vessel size, shape, and branching patterns, which are crucial for accurate segmentation. Experimenters should consider the distributions of any pathologies within the dataset, such as areas of stenosis or aneurysms, as these features may require specialized segmentation approaches. The statistical data also assist in assessing the heterogeneity of the patient population, ensuring that the segmentation method is robust across diverse patients. This thorough analysis is vital for refining and validating the segmentation method, yielding more reliable and clinically applicable results.

### Data preprocessing and augmentation

The preprocessing stage involves several essential steps: denoising, smoothing, resampling, and contrast enhancement. Denoising techniques, such as median filtering, are employed to reduce the image noise, minimize interference from low-quality images during segmentation, and improve the algorithm’s performance. Smoothing operations, such as Gaussian smoothing, eliminate discontinuous edges in the image and yield more continuous and recognizable blood vessel structures. Resampling ensures a standardized spatial resolution across images, reducing generalization errors caused by sampling device disparities and providing a consistent size and resolution for model learning and inference. Contrast enhancement techniques, such as histogram equalization or adaptive histogram equalization, improve the visibility of vascular structures in images. Specifically, we used mean filtering (kernel size: 3 × 3), Gaussian smoothing (standard deviation: 1, kernel size: 3 × 3), intensity normalization (*Z*-score), and adaptive histogram equalization (block sizes: 8 × 8, clip limit: 0.1) for preprocessing. In addition, since images in the same dataset had the same resolution (MLD: 0.264 mm × 0.264 mm × 0.8 mm, CSD: 0.4 mm × 0.4 mm × 0.4 mm), no resampling operation is performed.

Another critical step in the workflow is data augmentation. The dataset is expanded by applying various geometric transformations. These techniques, including image rotation, flipping, and intensity changes, increase the diversity and robustness of the data. In our experiments, we augment the data by flipping and rotating the images and adjusting their intensity to 0.9–1.1 times that of the original images. These transformations were introduced with a probability of 0.1 for each image during the training process.

### Model training

We implemented fourfold cross-validation at this stage. Specifically, we partitioned the data into training and test sets at an 8:2 ratio. The training set was divided into four equal subsets, with three subsets used as training data and one as validation data, for model training and validation. This process was repeated four times, employing different subsets as validation data in each iteration to encompass the entire training set. By using cross-validation, we can effectively utilize the limited dataset for training and validation, mitigating the risk of overfitting the model to a specific data distribution. Specifically, CSD encompassed 45 volumes; 36 were used for cross-validation (27 for the training set and 9 for the validation set), and 9 were used for testing. MLD encompassed 107 volumes; 85 were used for cross-validation (64 for the training set and 21 for the validation set), and 22 were used for testing. In this phase, we selected four network models for comparison training: U-Net [28], V-Net [29], UNETR [30], and SwinUNETR [31]. U-Net is a classic convolutional neural network that enhances segmentation accuracy through its encoder-decoder structure and skip connections. V-Net utilizes a residual network and multiscale residual module to capture fine details and contextual information. UNETR is a transformer-based model that leverages self-attention mechanisms to model pixel relationships effectively. SwinUNETR combines the Swin Transformer [32] and UNETR, where the Swin Transformer is a variant of the Transformer mechanism based on a local perceptual window. All the models used were three-dimensional segmentation models. During training, in the preprocessing process, a 192 × 192 × 64 sized image patch was cropped from the entire volume of 3D data for use as input to the models.

Due to the small proportion of cerebral vessels in the image, the foreground and background pixels are significantly imbalanced. We use a weighted combined variant of dice loss and focal loss [33] to address this imbalance and enhance cerebrovascular segmentation. The dice loss effectively handles the foreground–background pixel imbalance, while the focal loss focuses on hard-to-classify pixels by adjusting sample weights. By modifying the weights of the dice loss and focal loss and combining them using a weighted summation to form the final loss function, we balance their contributions to the segmentation results, leading to improved accuracy and robustness in cerebrovascular segmentation.

We utilized a workstation with 6 RTX 3090 GPUs, 2 Intel(R) Xeon(R) Silver 4310 CPUs, and 256 GB of RAM for model training and testing. The training process employed the AdamW optimizer with an initial learning rate of 0.0001 for 400 training epochs, utilizing a batch size of 1. To expedite model convergence and reduce training time, we also implemented a warmup strategy.

### Model validation

We evaluated the model using the validation set after every five rounds of each cross-validation fold. The entire image was predicted using a sliding window of size 192 × 192 × 64, and the dice similarity coefficient (DSC) was calculated as an internal performance metric. We then adjusted the model’s hyperparameters, such as the learning rate, network structure, or regularization parameters, based on the performance of the model on the validation set. We tested different combinations of hyperparameters and selected the model with the best performance on the validation set, i.e., the highest DSC.

We comprehensively compared the U-Net, VNet, UNETR, and SwinUNETR models using the CSD and MLD datasets and evaluated their performances based on several metrics: DSC, average surface distance (ASD), precision (PRE), sensitivity (SEN), and specificity (SPE).

### Postprocessing and analysis

Postprocessing was conducted to enhance the accuracy and quality of the image segmentation results. We employed operations such as edge smoothing, region merging, splitting, and filtering on every image in the validation set and test set. These operations fill voids and connect disjointed edges, merge small neighboring regions into more comprehensive areas, improve segmentation consistency and connectivity, and eliminate artifacts, isolated points, or mislabeling in the segmentation results. Specifically, we first performed mean filtering on the entire image and then removed regions smaller than 50 pixels and spliced neighboring regions that were no more than 10 pixels away from each other. Finally, we quantitatively and qualitatively evaluated the segmentation results by calculating various evaluation metrics on the test set and comparing them with the physician’s criteria for manual segmentation. The segmentation outcomes were visualized in three dimensions, enabling the feasibility and accuracy of the segmentation results to be observed.

### Statistical analysis

First, we calculated the performance metrics for each model and performed a Shapiro–Wilk test on the DSC, ASD, PRE, SEN, and SPE of the four models on the two test sets. If the *p* value exceeded 0.05, the data were normally distributed; if the *p* value was less than or equal to 0.05, we examined the quantile–quantile plot; and if the data were distributed around a straight line, we assumed that the data were normally distributed. Otherwise, the data were assumed to not follow a normal distribution. We then used one-way ANOVA to compare the performances of the different models. We calculated the *F*-statistic and the corresponding *p* value. A *p* value less than 0.05 was considered to indicate a statistically significant difference between the performances of the models. All analyses were performed using the IBM SPSS statistical software (version 27).

## Results

### Model performance

Table 2 presents the mean DSC metrics for the CSD and MLD validation sets. The variations in the DSC metrics of the four models in each fold validation were insignificant, suggesting that the data distribution does not strongly influence the segmentation workflow.

**Table 2:** Mean DSC metrics for the CSD and MLD validation set, with the best models in the four-fold cross-validation set highlighted in bold

Dataset	Fold	U-Net	V-Net	UNETR	SwinUNETR
CSD	Fold 0	0.688	0.826	0.825	0.860
	Fold 1	0.686	0.817	0.825	**0.864**
	Fold 2	0.679	0.819	0.816	0.857
	Fold 3	**0.704**	**0.826**	**0.832**	0.848
MLD	Fold 0	0.574	0.628	0.612	0.657
	Fold 1	0.597	**0.641**	**0.629**	**0.684**
	Fold 2	0.589	0.637	0.607	0.654
	Fold 3	**0.603**	0.622	0.625	0.664

*CSD* Cerebrovascular segmentation dataset, *MLD* Middle cerebral artery M1 segment with lenticulostriate artery segmentation dataset

**Fig. 2:**
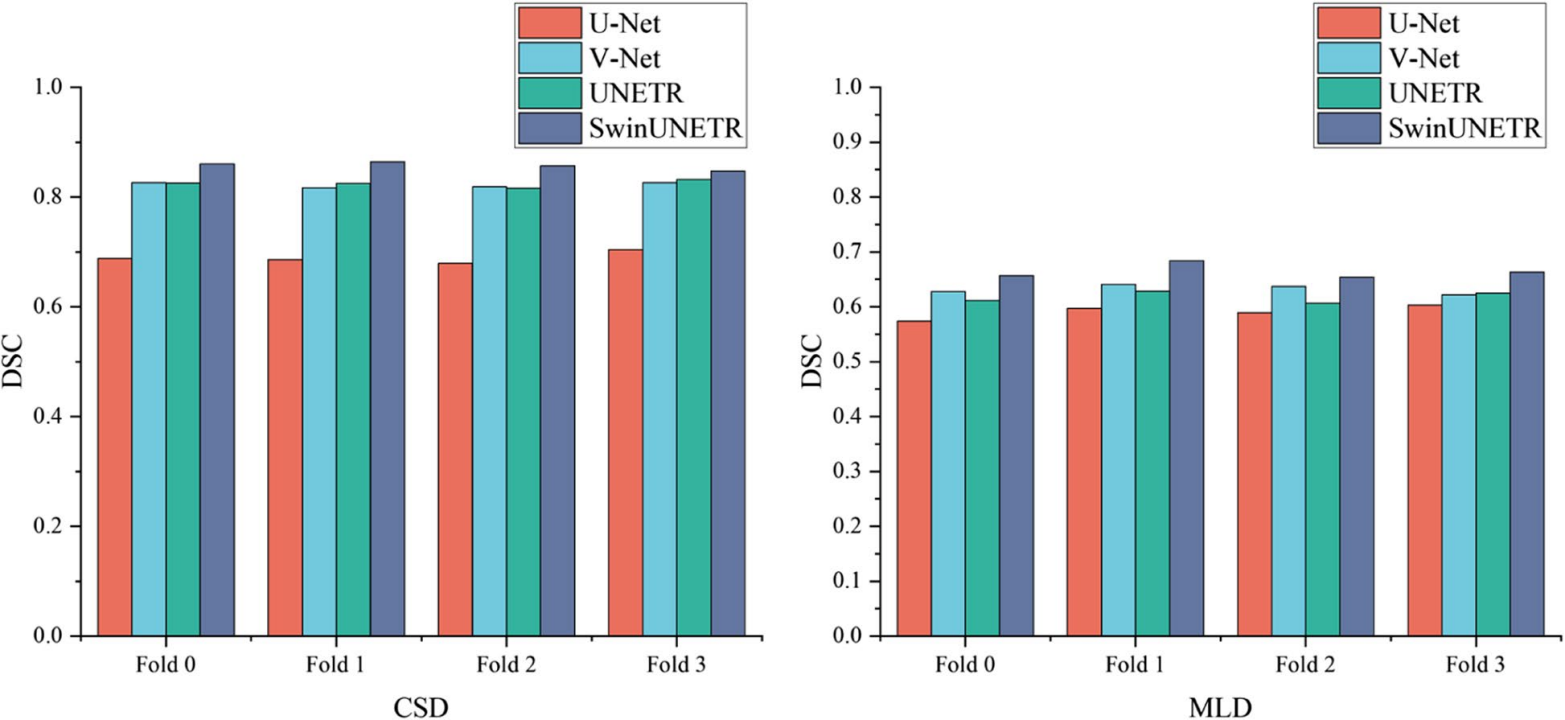
Mean DSC metrics for different models on two fourfold cross-validation sets

Figure 2 illustrates that SwinUNETR achieved the highest DSC in each fold validation, highlighting the significant potential of the Transformer structure for cerebrovascular segmentation tasks.

We recorded the results in Table 3 and 4 to illustrate the quantitative analyses these models on the two test sets. On the CSD test set, SwinUNETR outperformed the other models in the DSC, PRE, SEN, and SPE metrics, while V-Net exhibited the best performance in the ASD metric. On the MLD test set, SwinUNETR demonstrated an outstanding ability to segment cerebral vessels of different sizes despite not achieving the best MCA segmentation results. The proposed algorithm still excelled in the DSC, ASD, PRE, and SPE metrics for LSA segmentation. This finding indicates that SwinUNETR is capable of effectively handling diverse vessel sizes. Of course, V-Net also performed strongly, indicating that traditional CNNs still possess advantages in this task. Moreover, through statistical tests, we found that the models’ performance metrics on the test set conformed to a normal distribution. The performance metrics of the models significantly differed (*p* < 0.05) for all metrics except the DSC_M_ (*p* = 0.225), PRE_M_ (*p* = 0.325), and SPE_M_ (*p* = 0.063).

**Table 3:** Evaluation results of the four best validation models on the CSD test set; the best metrics are highlighted in bold

CSD	DSC	ASD	PRE (%)	SEN (%)	SPE (%)
U-Net	0.705	61.69	65.5	76.8	99.905
V-Net	0.817	**0.99**	80.7	82.9	99.953
UNETR	0.822	3.68	80.9	83.8	99.953
SwinUNETR	**0.853**	2.67	**84.8**	**86.0**	**99.963**

*CSD* Cerebrovascular segmentation dataset, *MLD* Middle cerebral artery M1 segment with lenticulostriate artery segmentation dataset, *DSC* Dice similarity coefficient, *ASD* Average surface distance, *PRE* Precision, *SEN* Sensitivity, *SPE* Specificity

**Table 4:** Evaluation results of the four best validation models on the MLD test set; the best metrics are highlighted in bold

MLD	DSC_M_	DSC_L_	ASD_M_	ASD_L_	PRE_M_ (%)	PRE_L_ (%)	SEN_M_ (%)	SEN_L_ (%)	SPE_M_ (%)	SPE_L_ (%)
U-Net	0.724	0.468	11.35	5.64	68.5	49.0	79.9	46.2	99.9906	99.9958
V-Net	**0.766**	0.482	**2.38**	4.34	73.6	39.2	**83.1**	**67.2**	99.9925	99.9902
UNETR	0.727	0.497	3.70	7.59	**77.0**	48.9	71.4	52.1	**99.9946**	99.9952
SwinUNETR	0.761	**0.562**	5.86	**3.45**	74.1	**56.0**	80.8	58.3	99.9928	**99.9960**

*CSD* Cerebrovascular segmentation dataset, *MLD* Middle cerebral artery M1 segment with lenticulostriate artery segmentation dataset, *DSC* Dice similarity coefficient, *ASD* Average surface distance, *PRE* Precision, *SEN* Sensitivity, *SPE* Specificity, *_M_ Metrics of middle cerebral artery M1, *_L_ Metrics of lenticulostriate artery

**Fig. 3:**
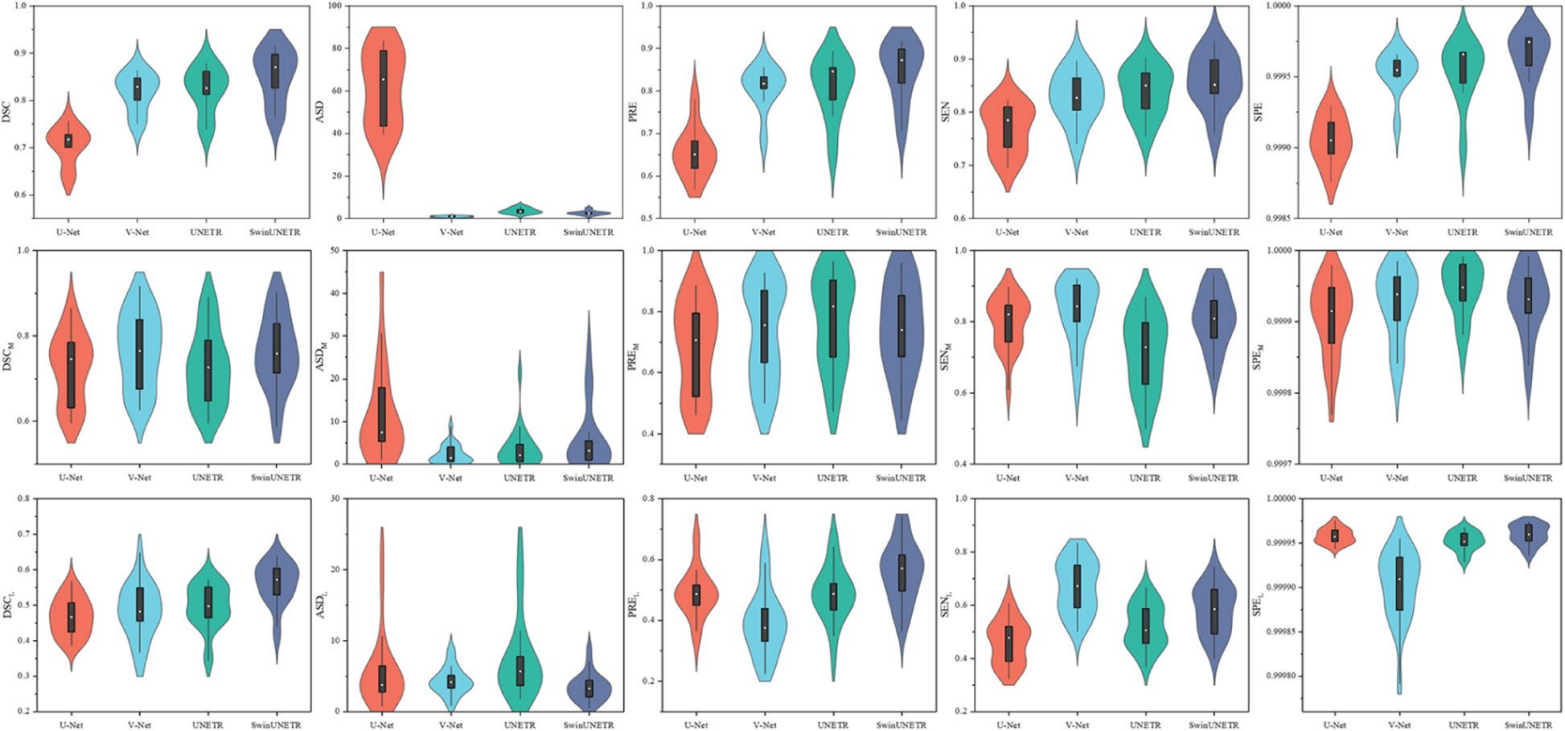
Distributions of the evaluation results of the four best-validated models on the test sets. The first row shows the results for CSD, and the second and third rows show the results for MLD

To further visualize the performance metrics of these models on the two test sets, the results are depicted in Fig. 3 for better observation. These results illustrate that V-Net achieves a relatively low ASD index, indicating its proficiency in capturing the edge information of cerebrovascular images. Furthermore, UNETR and SwinUNETR performed well in DSC, suggesting that the attention mechanism of the Transformer structure effectively captures contextual information and helps restore the cerebrovascular structure in the deep learning model.

### Segmentation visualization

**Fig. 4:**
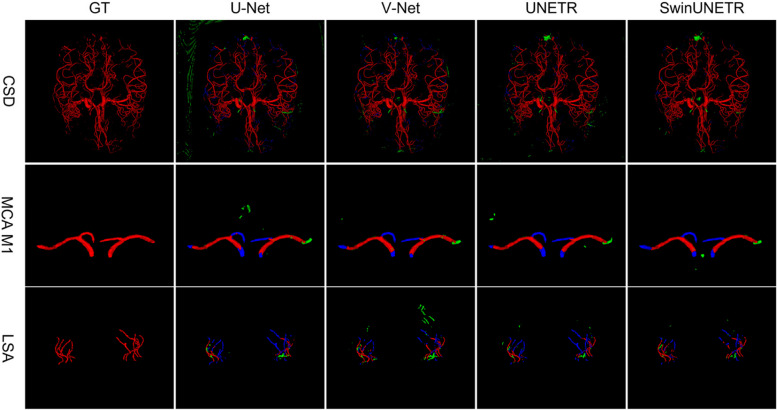
Visualizations of the ground truth and four model segmentation results. Red indicates true positives, green indicates false positives, and blue indicates false negatives. The first row shows the entire brain vessel in CSD, and the second and third rows show the middle cerebral artery (MCA M1) and the lenticulostriate artery (LSA) in MLD

To facilitate visual comparison of the segmentation effects of each model on the cerebral vasculature, we randomly selected one patient from each of the two test sets. Figure 4 visualizes the ground truth and segmentation results of the four models, encompassing the entire cerebral vasculature of the patient in CSD and the MCA M1 with the LSA of the other patient in MLD. Notably, SwinUNETR more accurately identified vessel areas, achieving significantly smaller areas of omission (blue) and misclassification (green).

## Discussion

The cerebrovascular segmentation task is challenging due to low contrast and presence of noise in medical images, coupled with complex vascular structures, data inhomogeneity, and labeling difficulties. While many articles have explored the potential of deep learning for cerebrovascular segmentation, they often concentrate on improving the accuracy or segmenting specific data sequences [34], neglecting the performance of the model on different datasets and vessels of different sizes. Therefore, we aimed to develop a deep learning cerebrovascular 3D segmentation workflow capable of handling multimodal clinical datasets, yielding accurate cerebrovascular structural segmentation results. Since this segmentation workflow can integrate different CNN and transformer models, for different multimodal sequence datasets, the workflow can use more targeted segmentation models to obtain better performance indicators. Our proposed workflow significantly boosts cerebrovascular segmentation accuracy, with SwinUNETR demonstrating superior performance across 4-fold cross-validation, achieving high DSC, precision, sensitivity, and specificity. Its impressive results on both the CSD and MLD test sets underscore the workflow’s practical value. This integrated approach provides a robust and reliable solution for cerebrovascular segmentation tasks.

This study specifically focused on validating the ability of the segmentation workflow across diverse clinical datasets. We used a public TOF-MRA dataset to achieve this goal and constructed a black blood sequence dataset of cerebrovascular images. In addition, we were among the first to segment the black blood sequence on MRI. We thoroughly explored the workflow’s performance and generalization capabilities under different scenarios through comprehensive experimental evaluations of both datasets.

We conducted a comparative analysis of various models and observed that deep learning-based workflows for 3D cerebrovascular segmentation yielded favorable results. The following indicators were used in the process: the DSC measures the degree of overlap between the segmentation results and the reference standard, with values ranging from 0 to 1; values closer to 1 indicate greater similarity between the segmentation results and the reference standard. The ASD calculates the average distance between the segmentation result and the reference standard, assessing the proximity between the result and the actual vessel boundary. Smaller ASD values indicate a closer approximation to the vessel boundary. Additionally, the PRE represents the ratio of the correctly classified positive samples in the segmentation result to the total number of samples classified as positive. Higher precision values imply that the model accurately identifies more vessel structures in the segmentation results. The SEN measures the ratio of correctly classified positive samples in the reference standard to the total number of positive samples. A higher recall rate indicates a stronger ability to effectively capture vascular structures. Finally, SPE evaluates the ratio of correctly classified negative samples to the reference standard’s total number of negative examples. A high specificity value indicates that the model can accurately exclude nonvascular regions. The results demonstrate that the models achieved different levels of performance according to the metrics used. Notably, V-Net excels in the ASD metric, indicating a strong ability to capture structural information about blood vessel edges. SwinUNETR, in contrast, exhibits strong performance in the DSC metric. These results highlight the distinct advantages and suitable scenarios of each model, emphasizing the importance of choosing the appropriate model considering the specific requirements of the situation. Furthermore, our workflow can seamlessly incorporate many deep learning models to address complex and dynamic clinical scenarios.

During the workflow evaluation, we observed that the deep learning models had relatively weaker segmentation ability for smaller vascular structures. While the CNN and Transformer models excel in capturing the spatial relationships and subtle features of cerebral blood vessels, thus enhancing the segmentation accuracy and detail retention of these models, their performance declines as the number of vascular structures is decreased. For instance, the DSC metric of the deep learning model for MCA M1 segmentation approaches 0.8. However, the DSC decreases to less than 0.6 on finer LSA segmentations. This phenomenon suggests that the segmentation capabilities of existing deep learning models for delicate structures can be improved.

Furthermore, we compare our results with the results of other studies. Wu et al. [24] proposed a weakly supervised cerebrovascular segmentation network that achieved a DSC of 0.831 in a public TOF-MRA dataset from the MIDAS data platform. Chen et al. [25] demonstrated the generative consistency of TOF-MRA-based semi-supervised cerebrovascular segmentation; their model’s best DSC was 0.788. Chen et al. [26] generated the publicly available CSD dataset and proposed a 3D adversarial network model called A-SegAN; this model achieved a DSC of 0.864 for cerebral vessel segmentation in TOF-MRA volumes. Our approach primarily differs from these methods because our approach has been validated on TOF-MRA and incorporates 3D T1 VISTA sequences; moreover, our approach considers the segmentation of both the entire cerebral vasculature and smaller specific vascular segments, such as the lenticulostriate arteries. On the CSD test set, our method obtained a DSC value of 0.853, which is slightly lower than the 0.864 of the A-SegAN [26]. This may be because they used only 5 data points for the test set, while we used 9. On the MLD test set, the DSC is 0.766 for MCA and 0.562 for LSA (surpassing the 0.34 achieved by Ma et al. using HighRes3DNet [35] because our approach uses a more advanced Transformer structure).

These findings reveal promising avenues and challenges for future research. First, enhancing workflow performance requires improving the network architecture. Although traditional network architectures have shown success, they still have untapped potential. Future research can explore novel model architectures or incorporate multiple models to improve the segmentation of fine-grained structures. For example, introducing an attention mechanism, increasing network depth and width, employing unconventional convolutional kernels, and incorporating generative adversarial network mechanisms [36] could all improve the network architecture. Second, the dataset must be expanded. Publicly available cerebrovascular datasets are limited due to confidentiality and the difficulty of accessing medical data. To address this, future research can focus on constructing larger publicly available cerebrovascular datasets by fostering multicenter cooperation, data sharing, and data synthesis. Finally, evaluation metrics and processes must be standardized. This standardization will aid in comparing and benchmarking various approaches.

## Limitations

This study has several limitations that should be acknowledged. First, the dataset used in this study may be limited in size and diversity. Although we attempted to utilize publicly available datasets, these datasets might only partially represent the vast variability of clinical scenarios and image qualities encountered in real-world situations. Second, while we employed standard evaluation metrics, these metrics may not fully encompass all aspects of segmentation performance. Additional metrics or criteria (e.g., manual scoring of segmentation quality) could provide a more comprehensive assessment. We did not include these metrics due to cost and efficiency issues and a more comprehensive assessment could be pursued in subsequent studies. Third, this study was primarily focused on MRI data and investigating the performances of the models on different MRI sequences. However, the performances of other imaging modalities (e.g., CTA) still need to be explored. Fourth, specific models (U-Net, V-Net, UNETR, and SwinUNETR) were compared in this study. While these models performed well, other state-of-the-art models may be worth investigating to potentially improve the proposed model. Finally, although the models exhibited good segmentation results, clinical validation through expert radiologist assessments or direct comparisons with ground truth manual segmentation results could provide additional insights into the reliability and accuracy of the models.

## Conclusion

In conclusion, we are the first researchers to segment black blood sequence MRI cerebrovascular images and explore the feasibility of deep learning for segmentation of the smallest cerebral vessels. Our proposed deep learning cerebrovascular 3D segmentation workflow accomplishes whole cerebrovascular segmentation on CSD. In addition, we compare MCA and LSA segmentation on MLD and investigate the performances of four deep learning models across bright and black blood MRA sequences as well as their abilities to segment cerebral vessels of varying sizes. The results demonstrate that the integrated workflow combining the CNN and Transformer models exhibits outstanding performance and capabilities, providing physicians with a powerful tool for visualizing vascular structures. This workflow contributes to the enhancement and applicability of cerebrovascular segmentation and fosters the application and development of deep learning in the cerebrovascular segmentation field.

## Data Availability

The datasets generated and analyzed during the current study are not publicly available but are available from the corresponding author on reasonable request.

## Abbreviations

3D T1 VISTA: 3.0 T three-dimensional T1-weighted sequence of volumetric isotropic turbo spin echo acquisition
ASD: Average surface distance
CNN: Convolutional neural network
CSD: Cerebrovascular segmentation dataset
DSC: Dice similarity coefficient
LSA: Lenticulostriate artery
MCA: Middle cerebral artery
PRE: Precision
SEN: Sensitivity
SPE: Specificity
TOF-MRA: Time-of-flight magnetic resonance angiography
